# Supplementary material for: How peer mechanism impacts loan repayment in a Self-help group?: An empirical study in India
Source: PLoS One. 2026 Jan 27;21(1):e0341674. doi: 10.1371/journal.pone.0341674 (PMC12843600; doi:10.1371/journal.pone.0341674)
Supplement: Attachment — Submitted filename: Comments by BRD_Sept 6 2025.pdf [file pone.0341674.s005.pdf]

How peer mechanism impacts loan repayment in a Self-help group? An empirical study

- **Topic:** Good, interesting and researchable. But the study location is missing. Better to add at the last of the topic – in India

- **Abstract** –Lengthy, concise it.

- **Introduction**: Poor. The contextual issues are missing. Use of old citations (not after 2017). Need to add latest citations. Remove problem statements and objectives of the research under the sub-headings (It is not a thesis, a research paper!). Keep it under the introduction part in a concise form.

- **Literature Review:** Poor. The theory, model and policy related to the research area is missing. Not shown the research gap and a conceptual framework. The review of paper is too old, not found citations after 2015. The way of citations is not proper. Significant improvement is required. Consult the papers and add the latest citations:

  Dhungana, B. R., Chapagain, R., Pokhrel, O. P., Sharma, L. K., & Gurung, J. B. (2025). Assessing the performance of microfinance institutions in Nepal. *Pacific Business Review International*, *17*(11).

  Dhungana, B. R., Chapagain, R., & Ashta, A. (2023). Alternative strategies of for-profit, not-for-profit and state-owned Nepalese microfinance institutions for poverty alleviation and women empowerment. *Cogent Economics & Finance*, *11*(2), 2233778.

  Chapagain, R. K., & Dhungana, B. R. (2020). Does microfinance affect the living standard of the household? Evidence from Nepal. *Finance India*, *34*(2).

Dhungana, B. R. (2016). Does loan size matter for productive application? Evidence from Nepalese micro-finance institutions. *Repositioning:The Journal of Business and Hospitality*, *1*, 63-72.

- **Methodology:** Poor. No need sub headings. Concise it and remove irrelevant writings.
- **Results and Discussion:** Poor. This section is missing. You're your results and analysis under this section. You need to compare your results with previous researchers that may be for and against with your findings. It is necessary to cite properly.
- **Conclusion:** Concise it. Highlight what interesting message you found and provide suggestions to the policy makers. Also highlight -why your research is useful for policy implications.
- **Others:** Follow the format as prescribed by the journal.

Major revision is required.

Reviewed: September 06, 2025
