# Supplementary material for: How peer mechanism impacts loan repayment in a Self-help group?: An empirical study in India
Source: PLoS One. 2026 Jan 27;21(1):e0341674. doi: 10.1371/journal.pone.0341674 (PMC12843600; doi:10.1371/journal.pone.0341674)
Supplement: Attachment — Submitted filename: RESPONSE TO REVIEWERS_PLOSONER1.docx [file pone.0341674.s007.docx]

**RESPONSE TO REVIEWERS**

1. **Addition of Recent and Relevant Citations:** As suggested by you, the comments have been included in the manuscript (Discussion section) and given as references 23, 27, 64, and 24. Citations suggested by the reviewer have been added
2. **Adherence to Journal Formatting Guidelines:**

The manuscript has been thoroughly revised, and all necessary changes have been implemented.

1) Reference style has been changed to Vancouver
2) Double spacing and line numbers have been added

I believe that these minor revisions have improved the overall clarity and scholarly contribution of the manuscript. We hope that the revised version meets the journal’s expectations and is now suitable for final acceptance.

3. Data has been anonymized and also an additional statement on ethical consideration and data collection has been added. The same has been highlighted under the Ethical consideration section
